# Improved Performance of All‐Solid‐State Lithium Metal Batteries via Physical and Chemical Interfacial Control

**DOI:** 10.1002/advs.202103433

**Published:** 2021-11-10

**Authors:** Jong Heon Kim, Kwangmo Go, Kyung Jin Lee, Hyun‐Suk Kim

**Affiliations:** ^1^ Department of Materials Science and Engineering College of Engineering Chungnam National University 99 Daehak‐ro, Yuseong‐gu Daejeon 34134 Republic of Korea; ^2^ Department of Chemical Engineering and Applied Chemistry College of Engineering Chungnam National University 99 Daehak‐ro, Yuseong‐gu Daejeon 34134 Republic of Korea

**Keywords:** all‐solid‐state lithium metal batteries, Li films, Li_6.25_La_3_Zr_2_Al_0.25_O_12_ (LLZO), plasma treatment, PVDF‐HFP, solid composite electrolytes, thermal evaporation

## Abstract

Lithium metal batteries (LMBs) show several limitations, such as high flammability and Li dendrite growth. All‐solid‐state LMBs (ASSLMBs) are promising alternatives to conventional liquid electrolyte (LE)‐based LMBs. However, it is challenging to prepare a solid electrolyte with both high ionic conductivity and low electrode–electrolyte interfacial resistance. In this study, to overcome these problems, a solid composite electrolyte (SCE) consisting of Li_6.25_La_3_Zr_2_Al_0.25_O_12_ and polyvinylidene fluoride‐*co*‐hexafluoropropylene is used, which has attracted considerable attention in recent years as a solid‐state electrolyte. To operate LMBs without an LE, optimization of the electrode–solid‐electrolyte interface is crucial. To achieve this, physical and chemical treatments are performed, i.e., direct growth of each layer by drop casting and thermal evaporation, and plasma treatment before the Li evaporation process, respectively. The optimized ASSLMB (amorphous V_2_O_5−_ *_x_* (1 µm)/SCE (30 µm)/Li film (10 µm)) has a high discharge capacity of 136.13 mAh g^−1^ (at 50 °C and 5 C), which is 90% of that of an LMB with an LE. It also shows good cycling performance (>99%) over 1000 cycles. Thus, the proposed design minimizes the electrode–solid‐electrolyte interfacial resistance, and is expected to be suitable for integration with existing commercial processes.

## Graphical abstract

The electrochemical properties of all‐solid‐state lithium metal batteries (ASSLMBs) are significantly improved through a novel combined fabrication method that involves physical and chemical processing steps. The optimized ASSLMB cell (amorphous V_2_O_5−_ *_x_* (a‐V_2_O_5‐X_)/solid composite electrolyte (SCE)/Li film) exhibits a high discharge capacity, which is 90% of that of a liquid‐based cell. It also demonstrates excellent cycling performance over 1000 cycles.

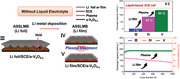

## 1 Introduction

Lithium metal batteries (LMBs) are used widely in various electronic devices, such as grid‐storage systems, portable electronics, and electric vehicles.^[^ ^1^, ^2^, ^3^, ^4^, ^5^, ^6^ ^]^ Accordingly, next‐generation LMBs must have characteristics such as high energy and power densities, fast charging, good cycling stability, and high safety.^[^ ^7^ ^]^ A key factor for achieving high‐performance LMBs is the development of advanced separators and electrolytes that can satisfy the operational requirements of high‐voltage cathode materials.^[^ ^8^, ^9^ ^]^ Therefore, it is necessary to develop electrolytes and separators that are nonflammable and have a large operating potential window and long‐term durability at high voltages. Solid‐state electrolytes (SSEs) meet these requirements.^[^ ^10^, ^11^, ^12^ ^]^ If high‐performance SSEs can be realized, this would solve all of the above‐mentioned problems simultaneously. Neither do SSEs contain flammable liquids nor do they suffer from dendrite formation at the anode; therefore, batteries with SSEs have greatly enhanced safety and cycling performance. Moreover, the use of high‐voltage cathode materials would be enabled by a high operating potential window. Therefore, all‐solid‐state lithium metal batteries (ASSLMBs) that do not use a liquid electrolyte (LE) are considered the ideal solution to the above‐mentioned problems.^[^ ^13^ ^]^

SSEs have been studied extensively, and previous reports on this topic have explored two types of materials: organic polymer electrolytes and inorganic ceramic electrolytes.^[^ ^14^, ^15^, ^16^, ^17^, ^18^, ^19^ ^]^ Both types of materials have distinct advantages and disadvantages. For instance, organic polymer electrolytes have highly tailorable characteristics and high interfacial stability with the electrodes, but are unstable at high voltages, suffer from Li dendrite growth (which degrades the durability), and have low ionic conductivity (<10^−4^ S cm^−1^) at room temperature. In contrast, inorganic ceramic electrolytes have excellent durability, high ionic conductivity, and a wide operating potential window, but are brittle and have poor interfacial stability. Recently, solid composite electrolytes (SCEs), which consist of ceramic fillers in a polymer matrix, have been proposed as effective SSEs because of their desirable physical and chemical properties, which are superior to those of single‐component electrolytes.^[^ ^20^, ^21^, ^22^, ^23^, ^24^, ^25^, ^26^ ^]^ Hence, there have been extensive efforts to develop high‐performance ASSLMBs by mixing various polymers and ceramic materials.^[^ ^27^, ^28^, ^29^ ^]^

Some studies have suggested that ASSLMBs are not completely in the solid state because some solvent residue is present in the SCE.^[^ ^30^, ^31^, ^32^ ^]^ In addition, it has been reported that the SCE introduces small amounts of LEs because of interfacial reactions.^[^ ^33^, ^34^, ^35^, ^36^, ^37^ ^]^ Therefore, although SCEs are being used in ASSLMBs, the high interfacial resistance between the electrode and solid electrolyte remains a limitation. This resistance originates from the fact that the components of ASSLMBs, viz. the cathode material, anode material, and solid electrolyte, must be fabricated individually. This results in the presence of a large number of voids being formed as a result of interfacial mismatches between the electrode and solid electrolyte, leading to a high interfacial resistance, which indicates poor diffusion of Li ions across the interface. Together, these factors degrade the electrochemical performance of ASSLMBs. Therefore, efforts are being made to develop ASSLMBs based on SCEs with improved properties to increase interfacial ionic conductivity.^[^ ^31^ ^]^ However, these strategies cannot prevent the formation of voids at the interface because the electrode and electrolyte materials are manufactured separately.

Hence, herein we propose a strategy to reduce the interfacial resistance between electrode and SCE based on physical and chemical treatment. To minimize the interfacial mismatch at the macroscale, Li metal was deposited by thermal evaporation to form a conformal thin film on the SCE. In addition, the surface of the SCE was modified by plasma treatment to increase the Li ion diffusion path between the Li metal and solid electrolyte, resulting in further reduction of the interfacial resistance. An amorphous V_2_O_5−_ *_x_* (a‐V_2_O_5−_ *_x_*) thin‐film cathode was fabricated on a stainless steel (SS) substrate using a radio frequency (RF) sputtering system. The a‐V_2_O_5−_ *_x_* thin film prepared at room temperature without post‐annealing had a similar or higher rate capability and better long‐term stability than other crystalline cathode materials (e.g., LiCoO_2_, LiMn_2_O_4_, and LiMn_1.5_Ni_0.5_O_4_).^[^ ^38^, ^39^, ^40^, ^41^, ^42^, ^43^, ^44^, ^45^ ^]^ Therefore, in this study, the a‐V_2_O_5−_ *_x_* thin film optimized in a previous study was used to manufacture high‐performance ASSLMBs.^[^ ^46^ ^]^ Then, a solid electrolyte was formed on the cathode by drop casting. Finally, the Li metal anode was directly deposited on the solid electrolyte by thermal evaporation. The SCE used in the ASSLMB was a polymer solid electrolyte (polyvinylidene fluoride‐*co*‐hexafluoropropylene or PVDF‐HFP) with good electrochemical stability and a ceramic solid electrolyte (Li_6.25_La_3_Zr_2_Al_0.25_O_12_; LLZO) with high ionic conductivity. The fabricated SCE had high ionic conductivity (4.2 × 10^−4^ S cm^−1^ at 80 °C), long‐term stability, and a wide operating potential range. Therefore, the proposed continuous growth process greatly reduced the interfacial resistance of the ASSLMB, resulting in improved electrochemical properties. As a result, a high‐performance ASSLMB was achieved with a capacity of 136.13 mAh g^−1^ (at 50 °C and 5 C) and excellent cycling performance over 1000 cycles. The proposed ASSLMB fabrication method effectively reduces the electrode–electrolyte interfacial resistance and is expected to be suitable for integration with existing commercial processes for fabricating LIBs.

## 2 Results and Discussion

**Figure 1:**
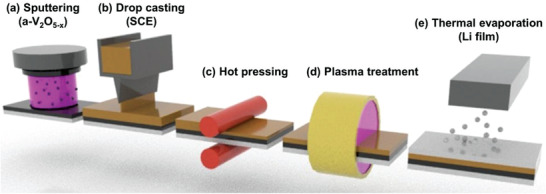
Schematic diagram of the process for fabricating a‐V_2_O_5−_ *_x_*/SCE/Li batteries: a) sputtering, b) drop casting, c) hot pressing, d) plasma treatment, and e) thermal evaporation.

**Figure** **1** schematically shows the proposed process for ASSLMB fabrication. In general, research groups fabricate each part of the SCE based ASSLMB individually and assemble the battery by applying pressure. The addition of an LE is crucial for reducing the interfacial resistance between the electrode and SCE.^[^ ^35^, ^37^, ^46^, ^47^, ^48^ ^]^ Here, we performed several processes, such as sputtering, drop casting, hot pressing, plasma treatment, and thermal evaporation in series to develop a continuous growth system that can be used for mass production of ASSLMBs at low temperature. First, an a‐V_2_O_5−_ *_x_* thin‐film with a thickness of 1 µm was formed on the current collector via RF sputtering for use as the cathode of the cell (Figure 1a). Next, the slurry of the SCE in *N*‐methyl‐2‐pyrrolidone (NMP), which contained LLZO powder, bis(trifluoromethane) sulfonimide lithium salt (LiTFSI), and PVDF‐HFP, was cast on the cathode to form a layer with a thickness of ≈60 µm (Figure 1b). Scanning electron microscopy (SEM) images of the SCE cross‐section before hot pressing are shown in Figure S1a (Supporting Information). The hot pressing process was performed simultaneously at 80 °C, which eliminated many of the voids in the SCE caused by the drop‐casting process, resulting in a film with a thickness of ≈30 µm (Figure 1c). Finally, we used plasma treatment (Figure 1d) and thermal evaporation (Figure 1e) to directly deposit a film of Li metal onto the SCE, resulting in few interfacial voids between the SCE and anode.

**Figure 2:**
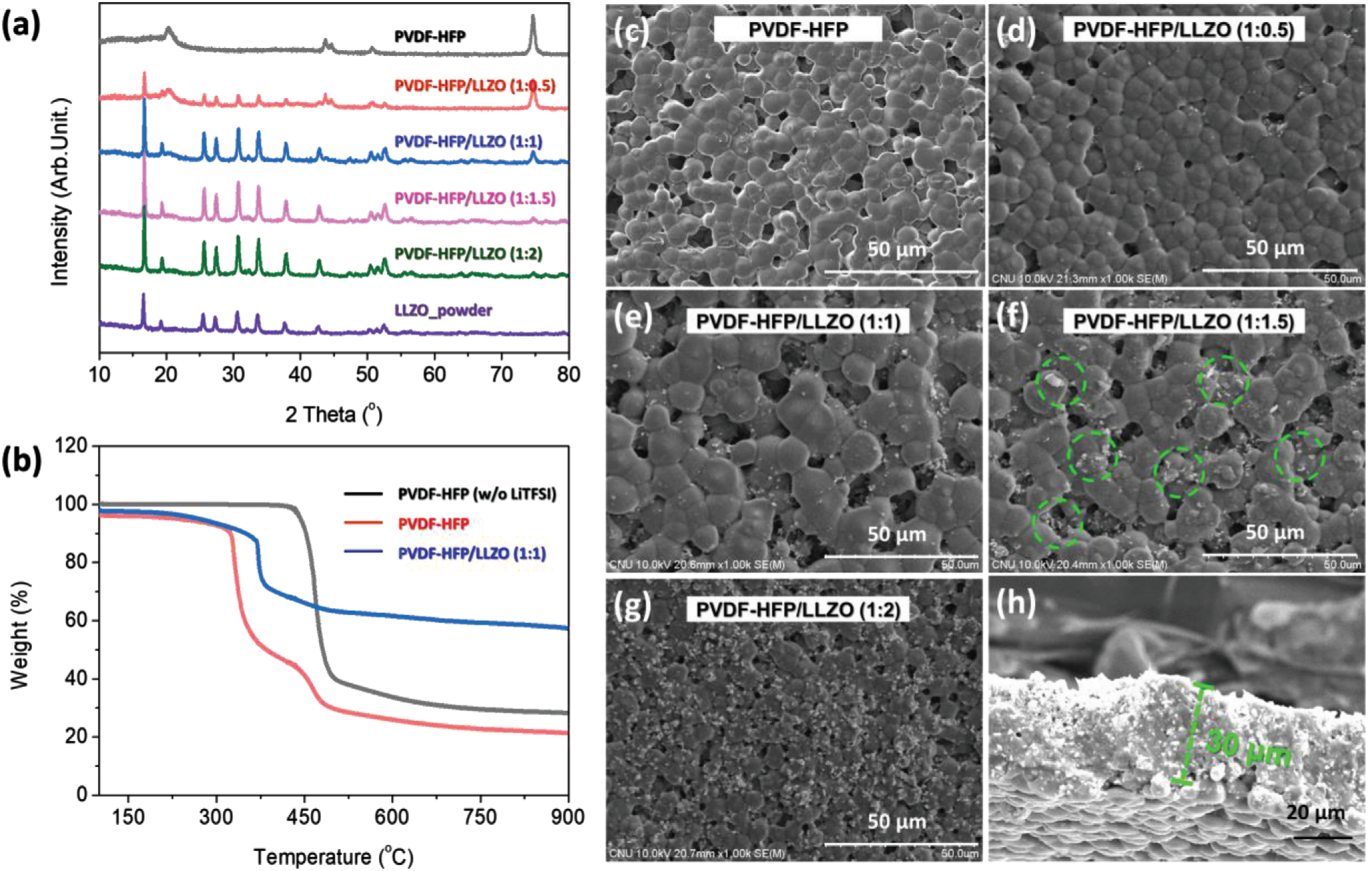
a) XRD patterns of SCEs with different PVDF‐HFP/LLZO ratios. b) TGA data for PVDF‐HFP (w/o LiTFSI), PVDF‐HFP, and PVDF‐HFP/LLZO. SEM images of SCEs with different PVDF‐HFP/LLZO ratios: c) pure PVDF‐HFP, d) 1:0.5, e) 1:1, f) 1:1.5, and g) 1:2. h) Cross‐sectional image of the SCE.

A solution of LLZO/PVDF‐HFP in NMP was sonicated and vortexed and used to prepare a homogeneous SCE layer consisting of LLZO and PVDF‐HFP. We used Al‐doped LLZO (cubic structure) in this study because of its high ionic conductivity. LLZO with a tetragonal structure has extremely low ionic conductivity. The crystallinity of the SCE was measured by X‐ray diffraction, as shown in **Figure** **2**a. The as‐prepared LLZO nanopowder showed peaks corresponding to the cubic structure of LLZO.^[^ ^49^ ^]^ Moreover, the intensities of these peaks in the pattern of the SCE increased with increasing fraction of LLZO in the SCE. The results of thermogravimetric analysis (TGA) of the PVDF‐HFP polymer, the polymer solution containing LiTFSI, and the SCE containing the polymer and LLZO in a 1:1 ratio yielded the degradation points and char yields of the PVDF‐HFP polymer, LiTFSI, and SCE, respectively (Figure 2b).

The SCE samples formed using different polymer‐to‐LLZO ratios were subjected to SEM analysis to examine their surface morphologies. Figure 2c,d–g shows the surface morphology of the PVDF‐HFP electrolyte and those of the SCE samples formed using the different PVDF‐HFP/LLZO ratios after drop casting, respectively. The crystals (marked by green circles) in the SEM images confirmed the presence of the LLZO nanopowder. The number of white crystals on the surface increased with increasing LLZO content (Figure 2d–g). Because the presence of a large number of voids in the SCE can adversely affect battery performance, the SCEs were hot pressed to reduce the number of voids, and also remove any residual solvent. Before hot pressing, the SCE surface morphology showed many voids (Figure S1b, Supporting Information). The PVDF‐HFP/LLZO (1:1 wt%) SCE had a thickness of 30 µm (Figure 2h), which is thinner than that of recently reported SCEs,^[^ ^33^, ^34^, ^50^, ^51^, ^52^ ^]^ and contained fewer voids.

**Figure 3:**
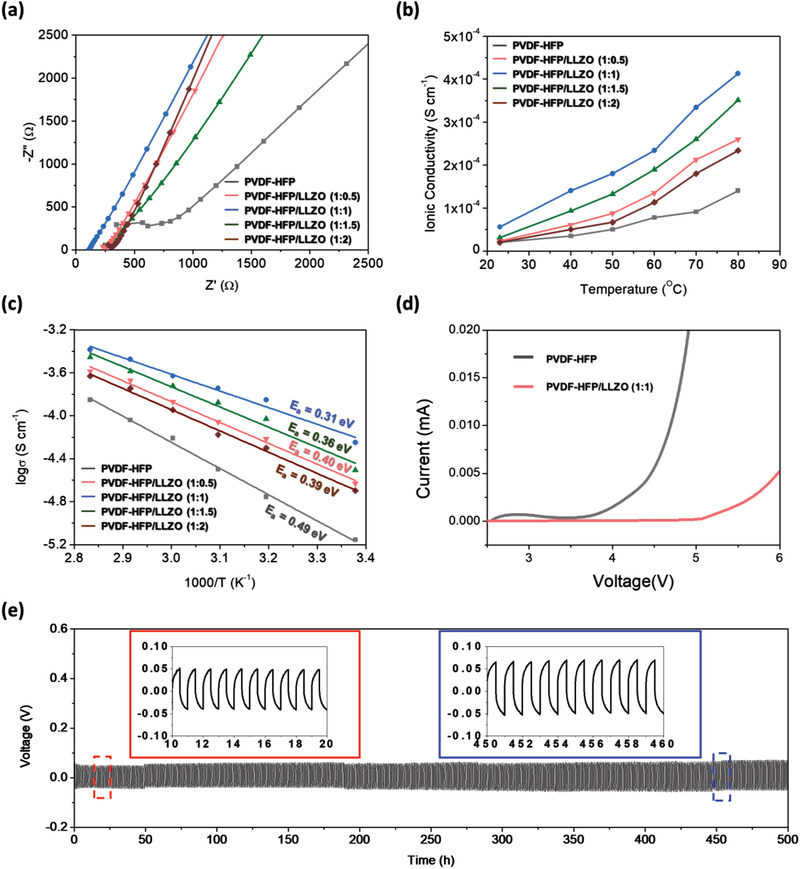
a) Impedance spectra of SCEs with different LLZO contents. b) Conductivity–temperature curves of SCEs with different LLZO contents and temperatures (23, 40, 50, 60, 70, and 80 °C). c) Arrhenius plots of various SCEs. d) Linear sweep voltammetry results for PVDF‐HFP/LLZO (1:1) and PVDF‐HFP. e) Voltage profiles for Li plating/stripping cycling process for the PVDF‐HFP/LLZO (1:1) SCE at current density of 0.5 mA cm^−2^.

The ionic conductivity of the solid electrolyte is one of the most important factors affecting the performance of ASSLMBs. Therefore, we used an SS/SCE/SS symmetric cell and subjected it to electrochemical impedance spectroscopy (EIS) while changing the LLZO content and temperature to investigate the Li^+^ conductivity ($\sigma_{Li^{+}}$) (**Figure** **3**a–c and Figure S2a–e, Supporting Information). Figure 3a shows the EIS results for SCE cells with different LLZO contents measured at 23 °C (room temperature; RT). The PVDF‐HFP/LLZO (1:1 wt%) sample showed the lowest impedance value at RT. In addition, Figure S2 (Supporting Information) shows the results of the EIS analysis at different temperatures (40–80 °C). The PVDF‐HFP/LLZO (1:1 wt%) sample showed the lowest impedance for the entire temperature range. The $\sigma_{Li^{+}}$ value was calculated using the following equation^[^ ^53^ ^]^

(1) $$\sigma_{Li^{+}}=\frac{d}{RA}$$

where *d* is the thickness (nm) of the SCE, *R* is the SCE resistance (Ω), and *A* is the area (cm^2^) of the metal contact. Figure 3b shows the $\sigma_{Li^{+}}$ values of each sample at various temperatures. For all samples, the calculated $\sigma_{Li^{+}}$ value increased with increasing temperature, regardless of the mixing ratio. The PVDF‐HFP/LLZO (1:1 wt%) sample had an ionic conductivity of 4.2 × 10^−4^ S cm^−1^ (at 80 °C), which was almost four times higher than that of PVDF‐HFP (1.1 × 10^−4^ S cm^−1^ at 80 °C). Moreover, this value is similar to the recently reported ionic conductivity for a composite electrolyte.^[^ ^33^, ^36^, ^50^, ^52^ ^]^ We determined the activation energy (*E*_a_) values for the ionic conductivity of the PVDF‐HFP/LLZO electrolytes using the Arrhenius empirical equation^[^ ^54^, ^55^ ^]^

(2) $$\sigma_{Li^{+}}=\frac{A}{T}\exp\left(-\frac{E_{a}}{k_{B}T}\right)$$

where *T* is the absolute temperature, *A* is the pre‐exponential constant, and *k*_B_ is Boltzmann's constant We determined *E*_a_ from the slope of log (*σ*) versus 1/*T* curves (Arrhenius plots) of the total conductivity of the SCEs with different compositions for the temperature range of 23–80 °C (Figure 3c). The PVDF‐HFP solid electrolyte had a relatively high *E*_a_ (0.49 eV), which suggested that it has a high Li‐ion migration barrier. In contrast, the PVDF‐HFP/LLZO (1:1 wt%) SCE showed a relatively low migration barrier (0.31 eV). In addition, the *E*_a_ values obtained in this study were similar to those reported previously for SCEs based on LLZO (0.28–0.47 eV).^[^ ^52^, ^54^, ^55^, ^56^, ^57^, ^58^ ^]^ These results indicate that the LLZO filler increases the ionic conductivity by reducing *E*_a_. However, the PVDF‐HFP/LLZO (1:1.5 wt%) and PVDF‐HFP/LLZO (1:2 wt%) samples had higher *E*_a_ values than that of PVDF‐HFP/LLZO (1:1 wt%). Thus, if not present in the optimal amount, the filler may interfere with Li ion diffusion in the SCE.

The electrochemical stability windows of the SCE samples were evaluated using the linear sweep voltammetry method for voltages of 2.5–6 V with Li/SCE/SS cells. PVDF‐HFP/LLZO (1:1 wt%) had good high‐voltage stability at voltages up to ≈5.0 V (vs Li/Li^+^) (Figure 3d). However, the window of electrochemical stability for PVDF‐HFP was limited to ≈3.8 V. This implies that the incorporation of LLZO as a nanofiller in the PVDF‐HFP matrix effectively promoted the electrochemical stability of the polymer electrolyte. Figure 3e shows that the Li/(PVDF‐HFP/LLZO (1:1 wt%))/Li cell showed a flat voltage profile and a low overpotential of ≈50–75 mV throughout the 500 h cycling process. This confirmed that the SCE had excellent stability, the interface between the SCE and Li metal was stable, and the formation of Li dendrites was prevented at a low current density of 0.5 mA cm^−2^ and temperature of 50 °C.

**Figure 4:**
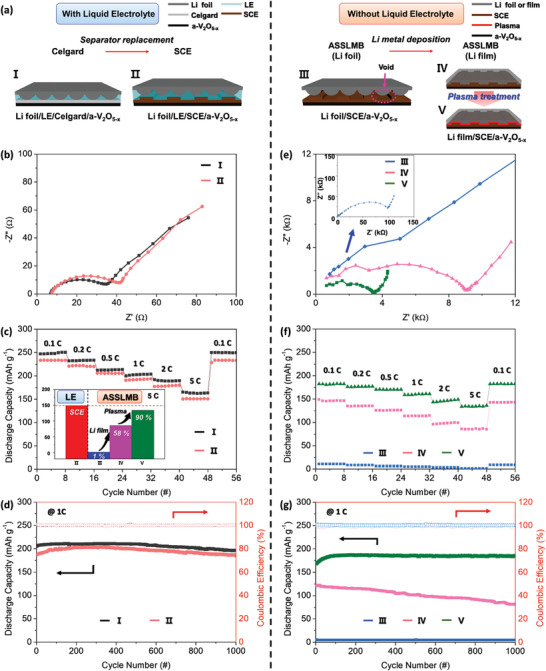
a) Schematic diagrams of structures of five cell types. Electrochemical properties of cells with LE: b) impedance spectra, c) rate capabilities at 0.1, 0.2, 0.5, 1, 2, and 5 C, and d) cycling performances at 1 C. Electrochemical properties of cells without LE: e) impedance spectra, f) rate capability, and g) cycling performance.

**Figure** **4** shows the electrochemical properties of an ASSLMB fabricated using the PVDF‐HFP/LLZO (1:1) electrolyte and a 1 µm a‐V_2_O_5−_ *_x_* cathode film. Several research groups have used Li metal foil as the anode when manufacturing SCE‐based ASSLMBs. However, the high interfacial resistance attributed to the interfacial mismatch between the Li foil and SCE is one of the biggest limitations of using Li foil. To overcome the high resistance induced by the interfacial mismatch, several previous studies added a small amount of an LE to the cells. Therefore, we compared the electrochemical characteristics of cells with different structures to reduce the interfacial resistance. Figure 4a shows a schematic diagram of the five types of cell structures analyzed in this study. The cell structures are categorized into two groups: with and without the use of an LE. Figure 4b–d shows the electrochemical properties of the cells containing a small amount of LE. Those of a conventional LMB structure with a commercial separator (Celgard 2400) are also shown for comparison. The initial galvanostatic charge/discharge curves were obtained at 0.1 C for voltages of 2.15–3.8 V (at 50 °C) for each cell, as shown in Figure S3a (Supporting Information). On comparing the charge/discharge curves for the LE‐based cells, it was found that the initial discharge capacities of the Li foil/LE/Celgard/a‐V_2_O_5−_ *_x_* (Type I) and Li foil/LE/SCE/a‐V_2_O_5−_ *_x_* (Type II) cells were 247.4 and 233.4 mAh g^−1^, respectively. Thus, the cells had similar discharge capacities. In addition, as shown in Figure 4b–d, both cells had similar electrochemical properties determined by EIS analysis, as well as similar rate capabilities and cycling performances. Additionally, the cycling performances showed a tendency to gradually decrease in both type‐I and type‐II cells. Therefore, the optimized SCE (PVDF‐HFP/LLZO (1:1)) had properties similar to those of the commercial separator, Celgard 2400.

Figure 4e–g shows the electrochemical performances of three types of LE‐free ASSLMBs. First, in keeping with previous research, we fabricated an ASSLMB by directly attaching a piece of Li foil to the SCE (type‐III cell). The use of the Li foil resulted in an impedance as high as ≈114 kΩ at 50 °C (inset of Figure 4e). This indicates that it is difficult to fabricate ASSLMBs with low interfacial resistance using Li foil if additional processing steps are not performed (e.g., the addition of an LE). Because of its high resistance, the type‐III cell showed poor charge/discharge characteristics (Figure S3b, Supporting Information). Therefore, as in the majority of previously reported studies, the use of a small amount of LE (about 20 µL) greatly reduced the interfacial resistance between the electrode and solid electrolyte, resulting in excellent electrochemical properties.^[^ ^33^, ^34^, ^35^, ^36^ ^]^ These studies showed similar electrochemical results to that of the type‐II cell prepared in this study. Therefore, to reduce the large interfacial resistance without adding an LE, Li metal deposition and plasma treatment of the SCE (as described in Figure 1) were sequentially applied. The ASSLMB with a Li film deposited by thermal evaporation (type‐IV cell) showed a significantly lower impedance (9 kΩ) than that of the type‐III cell (114 kΩ). Moreover, the type‐IV cell showed a discharge capacity of 147.0 mAh g^−1^ at 50 °C, which is ≈60% lower than that of the type‐I cell at 0.5 C. However, although the ASSLMB with the thermally evaporated Li film showed improved impedance characteristics, its electrochemical performance was less than ideal. Thus, the SCE surface was subjected to plasma treatment before the deposition of the Li film by thermal evaporation to further decrease the interfacial resistance between the Li anode and SCE (type‐V cell). As a result, the interfacial resistance was reduced by about 3 times, and this value is similar to the lowest impedance value (≈1 kΩ) of an ASSLMB without an LE reported previously.^[^ ^59^ ^]^ Furthermore, by fabricating Li/SCE/Li symmetric cells, the characteristics excluding the interfacial resistance between a‐V_2_O_5−_ *_x_* and SCE were analyzed. Figure S4 (Supporting Information) shows the results of impedance analysis similar to those of the a‐V_2_O_5−_ *_x_*/SCE/Li structure. These results indicate that the interfacial resistance between a‐V_2_O_5−_ *_x_* and SCE is negligibly small. And to evaluate the interfacial stability according to each cell through voltage profiles (Figure S5, Supporting Information). The type‐III, ‐IV, and ‐V cells showed excellent cycle stability for 200 h at a current density of 0.5 mA cm^−2^. In addition, all cells were analyzed according to different current densities (0.5, 1, 2, 5 mA cm^−2^). As a result, the voltage plateaus were stable without fluctuation in all cells. And then, among the three cells, the Li film with plasma treatment (type‐V) showed the lowest polarization voltage, which indicated the low interfacial resistance between the Li anode and the SCE.

Accordingly, as shown in Figure 4f, type‐V cells show much higher rate capability (136.13 mAh g^−1^ at 50 °C and 5 C) compared to type‐III and ‐IV cells. In addition, its cycling performance was superior to that of the type‐IV cell (Figure 4g). The type‐V cell showed excellent cycling performance over 1000 cycles without a deterioration in the discharge capacity. Therefore, it can be concluded that an interfacial plasma treatment is an effective way of enhancing the interfacial stability between Li and the SCE, resulting in improvements in the rate capability and cycling performance. In addition, the inset image in Figure 4c shows a comparison of the capacity properties of each battery structure formed using the SCE at 5 C. The optimized ASSLMB (type‐V cell) showed a capacity of 90% of that of the conventional LE‐based battery (type‐II cell). It is noteworthy that the type‐V cell used here had excellent cycling performance (>99% over 1000 cycles), comparable to (or even superior than) previous world records of ASSLMBs without LE additives (**Table** **1**).^[^ ^32^, ^59^, ^60^, ^61^, ^62^, ^63^ ^]^ Additionally, in Figure S6 (Supporting Information), high voltage drivability and electrochemical properties were compared after type‐II and type‐V structures were fabricated using NCM 622 thin‐film (1 µm) showing high voltage (>4 V) characteristics. As a result, charging and discharging proceed without decomposition of the SCE at high voltage, and the initial discharge capacity of type‐V cell is 140.1 mAh g^−1^, which represents 84.7% of the initial discharge capacity of type‐II cell. Moreover, similar to the case of using the aforementioned a‐V_2_O_5−_ *_x_* cathode, it shows a high rate capability of 90.8% compared to the type‐II cell at 0.5 C.

**Table 1:** Previously reported SCEs and comparison of their overall performances with that of optimized SCE fabricated in this work

Ref.	SCE	Cathode	Ionic conductivity [S cm^−1^]	Cut‐off potential [V]	Discharge capacity at 1st cycle [mAh g^−1^]	Capacity retention (cycles), C‐rate
^[^ ^59^ ^]^	LLZO/EmimFSI/PEO	NCM622	8.9 × 10^−5^ @ 25 °C	2.6–4.5 V @ 40 °C	145 (0.1C)	20% (15), 0.1C
^[^ ^60^ ^]^	LLZO/PEO/G4 /LiTFSI/BP	LFP	1 × 10^−4^ @ 40 °C	2.7–3.7 V @ 21 °C	163 (0.1C)	67% (200), 1C
^[^ ^61^ ^]^	PEO/PEG‐3LGPS	LFP	1.72 × 10^−3^ @ 50 °C	2.5–4.0 V @ 60 °C	168 (0.25C)	91% (150), 0.5C
^[^ ^62^ ^]^	PEO/MOF nanosheets/ LiTFSI	LFP	1.66 × 10^−5^ @ 25 °C	2.8–4.0 V @ 30 °C	130 (0.1C)	100% (50) 0.1C
^[^ ^63^ ^]^	PEO/PVDF/LiTFSII/Al_2_O_3_	LFP	1 × 10^−4^ @ 30 °C	3–3.9 V @ 50 °C	125 (0.1)	78% (410) 0.1c
This work	LLZO/PVDF‐HFP	a‐V_2_O_5−_ *_x_*	1.7 × 10^−4^ @ 50 °C	2.15–3.8 V @ 50 °C	145 (0.1C)	>99% (1000), 1C

**Figure 5:**
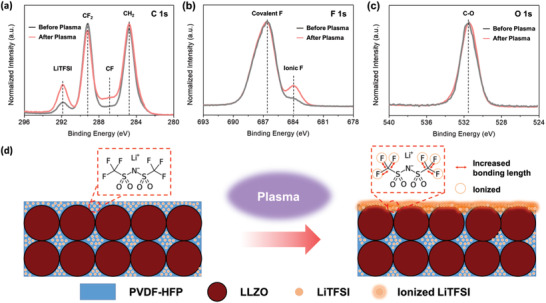
Effect of plasma treatment of the prepared SCE. a) C 1s, b) F 1s, and c) O 1s XPS spectra. d) Schematic illustration of the process.

It is surprising that the ASSLMB containing the SCE subjected to plasma treatment had a capacity that was 90% of that of an LMB with an LE. X‐ray photoelectron spectroscopy (XPS)‐based surface analysis was performed to investigate the effects of the plasma treatment on the SCE surface. The four peaks in the C 1s spectrum (**Figure** **5**a) at 284.8, 287.1, 289.2, and 291.9 eV were assigned to C—H, C—F, C—F_2_, and LiTFSI, respectively. After plasma treatment, the intensity of the peak related to LiTFSI increased. In addition, the plasma treatment induced differences in the F 1s spectrum (Figure 5b). For instance, the peak at the binding energy of 686.7 eV was related to the covalent F in LiTFSI and PVDF‐HFP, while the peak at 684.1 eV was related to the ionized and/or semi‐ionized F bonds. However, the peaks in the O 1s spectrum were not affected by plasma treatment; the only exception was the C—O peak at 531.8 eV (Figure 5c). These results indicate that the plasma treatment etched the SCE surface, thus exposing the LiTFSI salt on the surface. When the exposed LiTFSI salt came in direct contact with the Li film, the Li ions did not directly pass through the polymer matrix with low ionic conductivity, but moved to the LiTFSI salt region with ionic conductivity. In addition, the increase in the amount of ionized and/or semi‐ionized fluorine improved the interfacial adhesion between the SCE and neutral Li metal in the Li film. In other words, the plasma treatment etched the surface of the SCE, exposing the LiTFSI salt and ionizing the fluorine atoms. The effect of this plasma treatment is depicted in the schematic diagram of Figure 5d. This, in turn, reduced the interfacial resistance between the SCE and the Li film, resulting in improved electrochemical properties of the ASSLMB. A similar increment in the amount of ionic F present was observed in the case of the polymer/LiTFSI films (without LLZO) (Figure S7, Supporting Information). Furthermore, we analyzed the difference in the interfacial properties of the SCE/Li film with and without plasma treatment through the FIB‐SEM cross‐sectional image in Figure S8 (Supporting Information). It indicates that the physical adhesion between the interfaces is improved after plasma treatment. However, in Figure S9 (Supporting Information), no significant morphological differences were observed on the surface of the SCE after plasma treatment through SEM surface analysis. These results show that the strategy proposed here is highly suitable for producing liquid‐free solid electrolytes. In addition, because the amount of Li metal can be precisely controlled based on the cathode capacity, the overall cost can be reduced by limiting the amount of Li metal used.

These ASSLMB fabrication processes can also be extended to a different architecture. Therefore, while state‐of‐the‐art battery structures should be investigated in the future to evaluate the practical applicability of the proposed method, we examined the battery performance of an a‐V_2_O_5−_ *_x_*/Celgard system with LE/Li foil using ASSLMB using an SCE subjected to our proposed interfacial control process. To the best of our knowledge, this is the first report of a Li‐film‐based ASSLMB that shows better electrochemical performance than conventional SCE‐based ASSLMBs based on Li foil.

**Figure 6:**
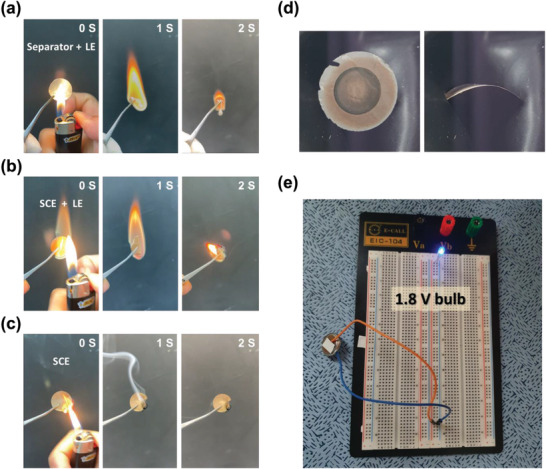
Flammability test of a) a separator or b) an SCE immersed in LE, and c) the SCE fabricated in this study. d) Front and side photographs of a‐V_2_O_5−_ *_x_*/SCE/Li cell showing its flexible nature. e) photograph of 1.8 V blue LED lit using a‐V_2_O_5−_ *_x_*/SCE/Li cell.

In addition, because we used SCEs, the fabricated ASSLMBs have several advantages over conventional separator/electrolyte structures, such as high flexibility and nonflammability. **Figure** **6**a shows the results of a fire‐retardant test of Celgard 2400 containing a small amount of an LE. The conventional Celgard 2400/LE structure was flammable and burned when exposed to an external fire. Moreover, the SCE with a small amount of the same LE also showed similar results (Figure 6b). However, the SCE fabricated in this study was not flammable (Figure 6c). The initial flame extinguished itself after 1 s, indicating a significant increase in the fire safety (Movies S1–S4, Supporting Information). Therefore, the SCE without an LE is a much safer ASSLMB, because the risk of thermal runaway is reduced with a nonflammable SCE. Figure 6d shows front (left) and side (right) images of the ASSLMB. The bendable SCE (Figure S10, Supporting Information) was also used in an LMB, thus confirming that it can be used to fabricate ASSLMBs. To evaluate the operation of the ASSLMB, a blue light‐emitting diode (LED) with an operating voltage of 1.8 V (Figure 6d) was tested. A single ASSLMB was sufficient to light the LED.

## 3 Conclusion

We developed a method for fabricating high‐performance ASSLMBs via an innovative processing strategy. The SCE used contains both LLZO and PVDF‐HFP to ensure high ionic conductivity, long‐term stability, and a high operating voltage. The a‐V_2_O_5−_ *_x_* cathode material was deposited by RF sputtering, while the Li metal anode film was deposited by thermal evaporation. In addition, the interfacial resistance between the Li metal and SCE was reduced by subjecting the SCE surface to a plasma treatment. The optimized ASSLMB, in which the interface between the electrode and the solid electrolyte was physically controlled (by direct growth of each layer) and chemically modified (by plasma treatment), showed desirable electrochemical properties. The high‐performance ASSLMB had a capacity only slightly below that of a similar cell structure using an LE. In addition, the optimized ASSLMB had excellent cycling performance (>99%) over 1000 cycles. Therefore, the continuous direct growth method described here can effectively improve the electrochemical properties of ASSLMBs and is expected to aid the development of next‐generation batteries.

## 4 Experimental Section

### Synthesis of PVDF‐HFP/LLZO Solid Electrolyte

NMP (Sigma‐Aldrich), LiTFSI (Sigma‐Aldrich), PVDF‐HFP (Mw = 450 000, Sigma‐Aldrich), and LLZO (particle size ≤ 500 nm; MSE Supplies) were used as purchased without further purification. PVDF‐HFP and LiTFSI were dissolved in NMP in a 2:1 weight ratio and sonicated until a homogeneous solution was formed. Next, LLZO was added to this solution in a PVDF‐HFP/LLZO weight ratio of 1:0.5, 1:1, 1:1.5, or 1:2. The solution was mixed with a vortex mixer (VM‐96B, Jeio Co. Ltd.) until it became homogeneous and was then cast onto the cathode. The SCE membrane was obtained after drying the cathode at 80 °C for 24 h to remove the NMP.

### Synthesis of a‐V_2_O_5−_ *_x_*/SCE/Li Cell

a‐V_2_O_5−_ *_x_* cathodes (diameter = 11 mm, thickness = 1 µm) were prepared by conventional on‐axis RF magnetron sputtering. A 99.99%‐pure V_2_O_5_ target (≈7.6 cm diameter) was used to fabricate all films. An RF power of 80 W (1.75 W cm^−2^) was applied at an ambient chamber pressure of 5 × 10^−6^ Torr. The working distance between the target and substrate was 54 mm. The a‐V_2_O_5−_ *_x_* cathode was deposited on an 304 type SS substrate (diameter: 16 mm) coated with layers of Ti (thickness = 20 nm) and Pt (thickness = 300 nm) (Pt/Ti/SS substrate).^[^ ^64^, ^65^ ^]^ The Pt/Ti layers on the SS substrate were pre‐sputtered before the deposition of the a‐V_2_O_5−_ *_x_* cathode by direct‐current sputtering using a power of 20 W (0.99 W cm^−2^) and a working distance of 50 mm under a working pressure of 3 × 10^−6^ Torr. The deposition was performed in a pure Ar atmosphere at RT. The Ti layer was applied to increase the adhesion between the Pt and SS, while the Pt layer acted as the current collector. Plasma treatment was performed at Harrick plasma, US PDC‐323‐2, Plasma Cleaner (150W, 75A, 50Hz). All processes were performed under 0.67 Pa and before treat 16 cc of air gas was fed into the chamber via a flow controller before ionized by the microwave radiation. After ionized air gas, the surface of SCE was treated for 5 min. Next, using thermal evaporation, the Li metal anode (thickness = 10 µm) and Cu current collector (thickness = 1 µm) were deposited on the SCE sequentially in the same chamber. During the Li and Cu evaporation processes, the pressure was maintained at 2 × 10^−6^ Torr. An air plasma treatment was performed on the SCE surface for 5 min under low vacuum (less than 5 × 10^−2^ Torr) to improve the contact and interfacial stability with the Li anode. Between each process step, the samples were kept in an Ar‐filled glove box (H_2_O <1 ppm, O_2_ <0.1 ppm) to prevent exposure to ambient air.

### Structural and Electrochemical Characterization

The morphology of the SCE was analyzed by scanning electron microscopy (SEM, S‐4800, Hitachi). Energy‐dispersive X‐ray spectroscopy (EDS) mapping was performed using a Merlin Compact (Zeiss) system. XRD analysis was performed using a D8 Discover (Bruker) system over the 2*θ* range of 10–80°. XPS was performed using a monochromatic Al K*α* X‐ray source to study the chemical bonding states. The peak positions were calibrated with respect to the C 1s peak (C—H_2_ = 284.8 eV). TGA was performed in a N_2_ atmosphere at a heating rate of 10 °C min^−1^ and 100–900 °C using a TGA N‐1000 (SINCO) system. To evaluate the electrochemical properties of the fabricated components, 2032‐type coin cells were assembled in an Ar‐filled glove box. All cells comprised the active material (electrode diameter = 11 mm) as the working electrode, bulk Li foil and a Li film as the counter electrode, a Celgard 2400 separator, the SCE, and an LE (1.0 M LiPF_6_). A 1:1 (vol) mixture of ethylene carbonate and diethylene carbonate (EC/DEC, 1:1 (v/v), Soulbrain Co. Ltd.) was used as the electrolyte. Galvanostatic charge/discharge tests were performed with a battery tester (WBCS‐3000, Wonatech) at constant current densities ranging from 0.1 to 5 C (1 C = 7.315 µA cm^−2^) for the voltages of 2.15–3.8 V (vs Li^+^/Li) at 50 °C. A sandwich cell with a SS/SCE/SS structure was used to measure the ionic conductivity using a one‐channel potentiostat (ZIVE SP1, Wonatech, Republic of Korea) within the frequency range of 1 Hz to 1 MHz at temperatures of 23, 40, 50, 60, 70, and 80 °C. Linear sweep voltammetry (LSV) experiments were performed using Li/SCE/SS cell. The experiments were conducted to investigate the electrochemical stability window of SCE. The potential range was scanned between open‐circuit potential and 6 V (vs Li^+^/Li) at a rate of 0.1 mV s^−1^. All electrochemical measurements were performed in an oven (OF‐12GW, JEIO TECH, Republic of Korea) to control the operating temperature. A blue LED was powered using a single a‐V_2_O_5−_ *_x_*/SCE/Li cell to test the performance of the fabricated ASSLMB.

## Conflict of Interest

The authors declare no conflict of interest.

## Supporting information

Supporting Information

Supplemental Movie 1

Supplemental Movie 2

Supplemental Movie 3

Supplemental Movie 4

## Data Availability

Research data are not shared.
