# Supplementary material for: Improved Performance of All‐Solid‐State Lithium Metal Batteries via Physical and Chemical Interfacial Control
Source: Adv Sci (Weinh). 2021 Nov 10;9(2):2103433. doi: 10.1002/advs.202103433 (PMC8805574; doi:10.1002/advs.202103433)
Supplement: Supplementary file 1 — Supporting Information [file ADVS-9-2103433-s003.pdf]

© 2021 Wiley-VCH GmbH

ADVANCED SCIENCE
Open Access

Supporting Information

for *Adv. Sci.*, DOI: 10.1002/advs.202103433

Improved Performance of All-Solid-State Lithium Metal Batteries via Physical and Chemical Interfacial Control

Jong Heon Kim, Kwangmo Go, Kyung Jin Lee, and Hyun-Suk Kim**

Supporting Information

Improved Performance of All-Solid-State Lithium Metal Batteries via Physical and Chemical Interfacial Control

Jong Heon Kim, Kwangmo Go, Kyung Jin Lee*, and Hyun-Suk Kim*

J. H. Kim, H.-S. Kim
Department of Materials Science and Engineering, College of Engineering, Chungnam National University, 99 Daehak-ro, Yuseong-gu, Daejeon 34134, Republic of Korea

K. Go, K. J. Lee
Department of Chemical Engineering and Applied Chemistry, College of Engineering, Chungnam National University, 99 Daehak-ro, Yuseong-gu, Daejeon 34134, Republic of Korea

*Corresponding authors: kjlee@cnu.ac.kr (K. J. Lee); khs3297@cnu.ac.kr (H.-S. Kim)

Experimental Section

Preperation of NCM622 thin-film: NCM622 cathodes (diameter = 11 mm, thickness = 1 μm) were prepared by conventional on-axis RF magnetron sputtering. A 99.99%-pure $LiNi_{0.6}Co_{0.2}Mn_{0.2}O_2$ target (~7.6 cm diameter) was used to fabricate all films. An RF power of 80 W (1.75 W cm^{-2}) was applied at an ambient chamber pressure of 5×10^{-6} Torr. The working distance between the target and substrate was 54 mm. The NCM622 cathode was deposited on an 304 type SS substrate (diameter: 16 mm) coated with layers of Ti (thickness = 20 nm) and Pt (thickness = 300 nm) (Pt/Ti/SS substrate). Post-annealing of NCM622 cathode was conducted at a temperature of 700 °C in a box furnace for 2 h.

Structural and Electrochemical Characterization: Cross-sectional FIB-SEM specimens were prepared by in situ lift-out in a dual-beam focused ion beam system (FIB; FEI Helios NanoLabTM).

Table of Contents

Figure S1. SEM images of SCE before hot pressing: (a) Cross-sectional image, (b) surface image.

Figure S2. Impedance spectra of SCE according to different LLZO content. (a) 40 °C, (b) 50 °C, (c) 60 °C, (d) 70 °C, and (e) 80 °C

Figure S3. Charge/Discharge curves. (a) with liquid electrolyte cells, and (b) without liquid electrolyte cells.

Figure S4. Impedance spectras of symmetric cells

Figure S5. (a) Voltage profiles of the symmetric cells at different current densities (0.5, 1, 2, and 5 mA cm^{-2}). (b) Voltage profiles of the symmetric cells at 5 mA cm^{-2}.

Figure S6. Electrochemical properties of two cell types with NCM622 thin-film: a) Charge/Discharge curves, and b) rate capabilities at 0.1, 0.2, 1, 2, and 5 C.

Figure S7. XPS spectrum of PVDF-HFP/LLZO (a-c), PVDF-HFP (d-f), and PVDF-HFP (w/o LiTFSI) (g-i).

Figure S8. FIB-SEM cross-section images of a) without plasma treatment and b) with plasma treatment.

Figure S9. The surface SEM images of a, b) before plasma treatment SCE and c, d) after plasma treatment SCE.

Figure S10. Photograph of flexible and bendable SCE.

Movie S1. Flammability test of separator.

Movie S2. Flammability test of separator immersed in LE.

Movie S3. Flammability test of SCE immersed in LE.

Movie S4. Flammability test of SCE.

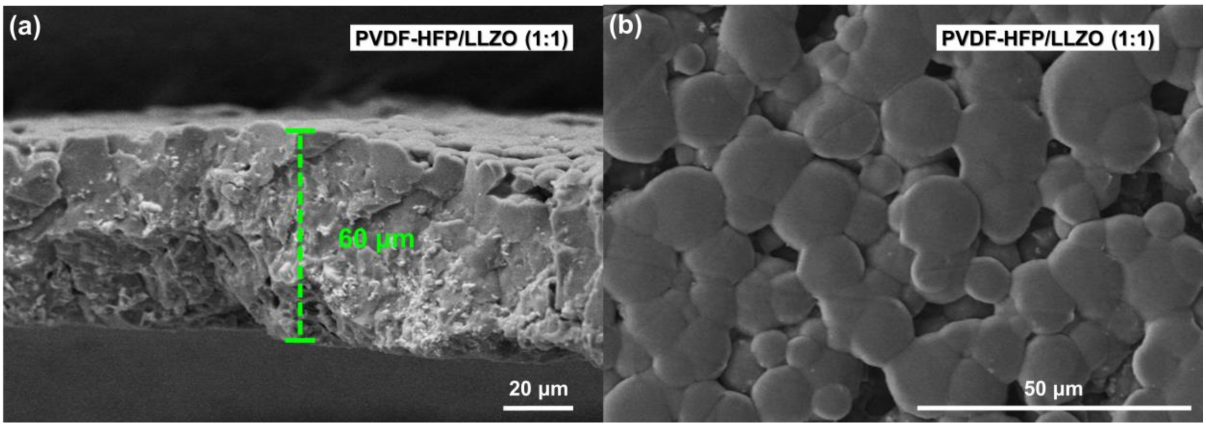

Figure S1. SEM images of SCE before hot pressing: (a) cross-sectional image, (b) surface image.

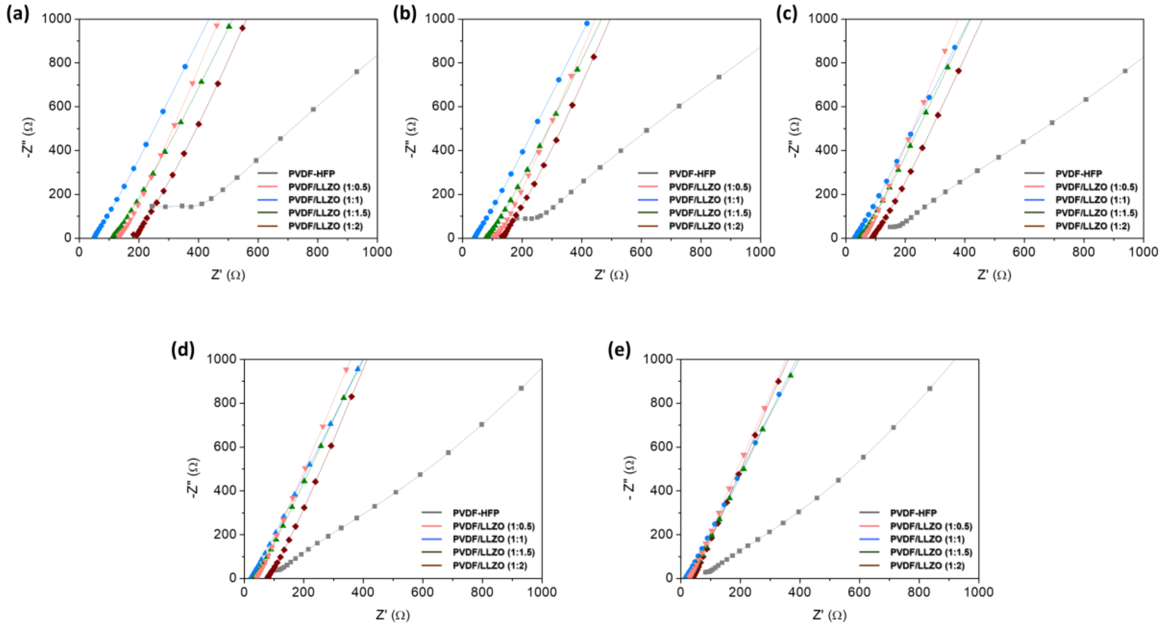

Figure S2. Impedance spectra of SCE according to different LLZO content. (a) 40 °C, (b) 50 °C, (c) 60 °C, (d) 70 °C, and (e) 80 °C

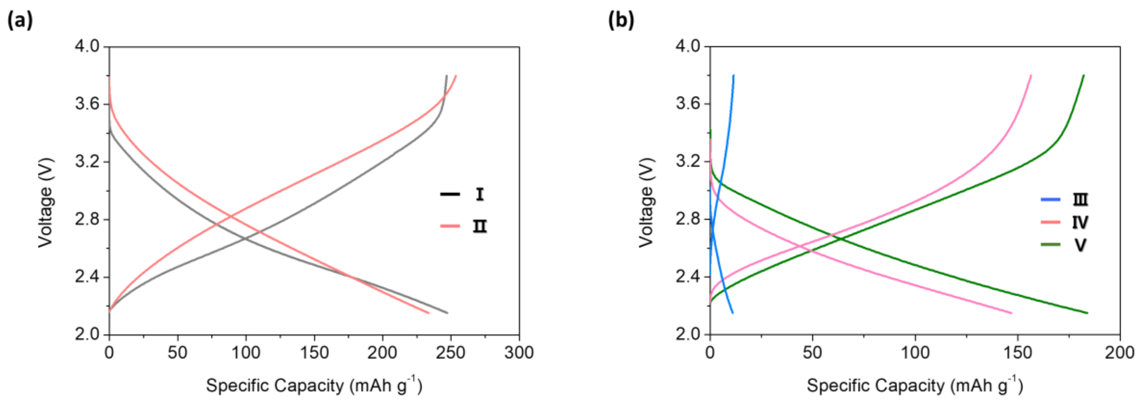

Figure S3. Charge/Discharge curves. (a) with liquid electrolyte cells, and (b) without liquid electrolyte cells.

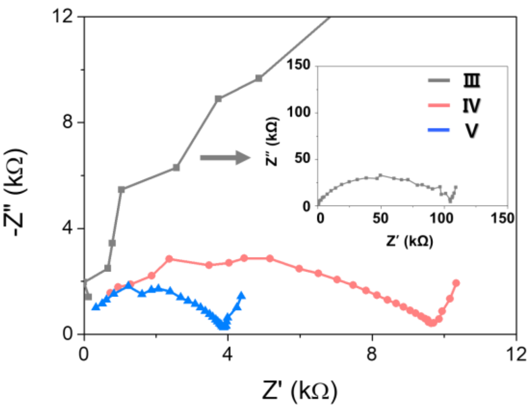

Figure S4. Impedance spectras of symmetric cells

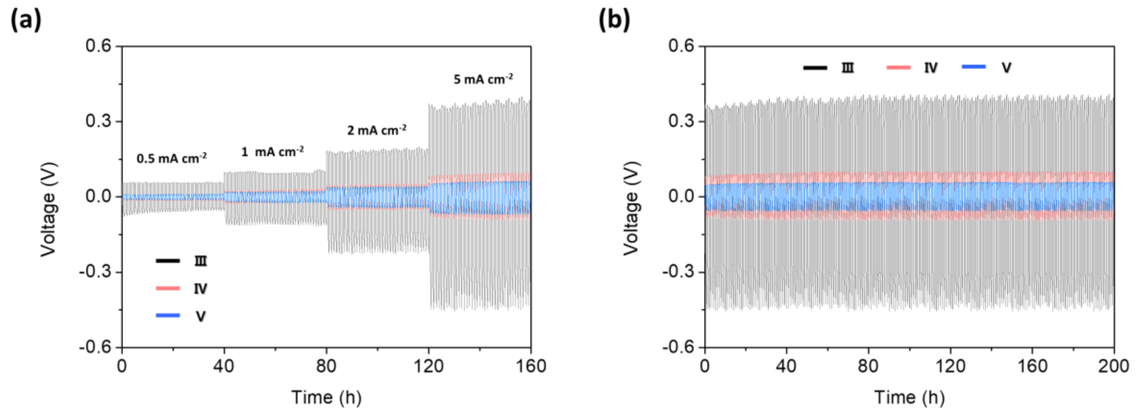

Figure S5. (a) Voltage profiles of the symmetric cells at different current densities (0.5, 1, 2, and 5 mA cm^{-2}). (b) Voltage profiles of the symmetric cells at 5 mA cm^{-2}.

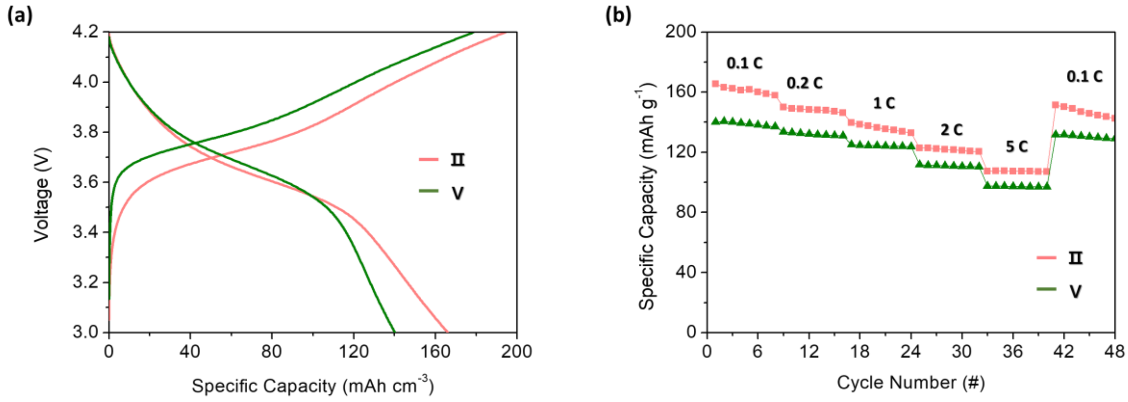

Figure S6. Electrochemical properties of two cell types with NCM622 thin-film: a) Charge/Discharge curves, and b) rate capabilities at 0.1, 0.2, 1, 2, and 5 C.

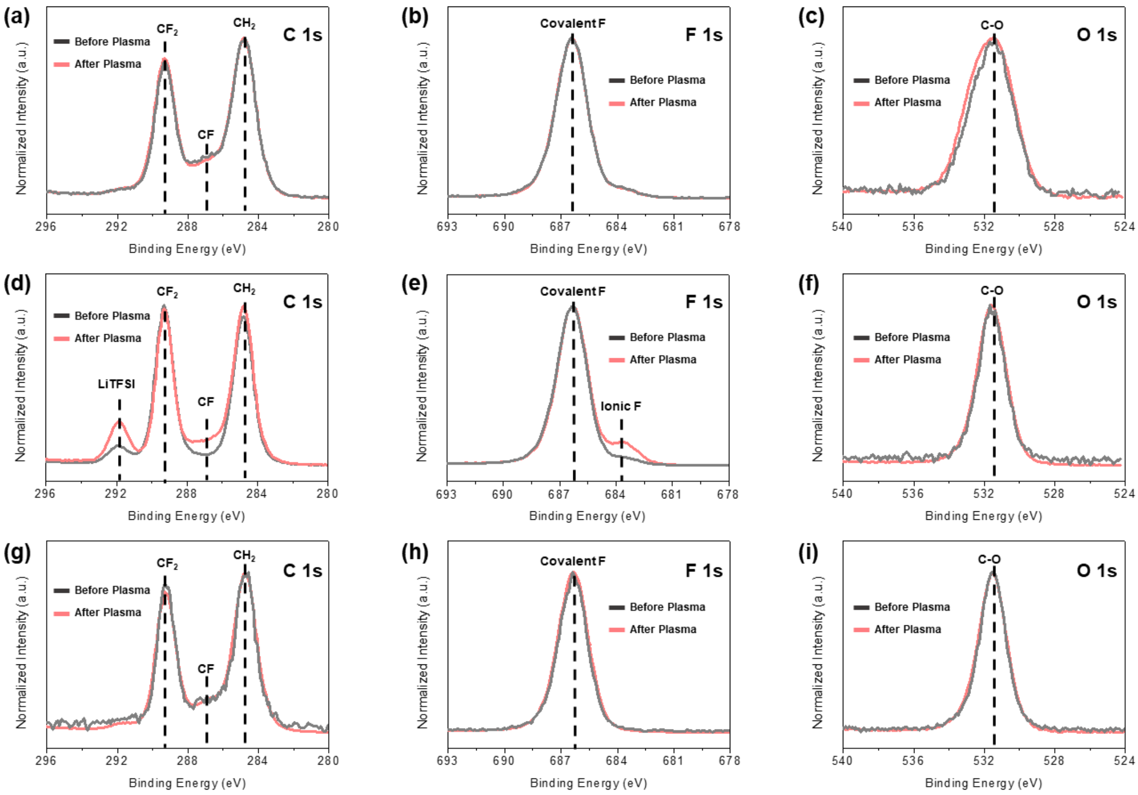

Figure S7. XPS spectrum of PVDF-HFP/LLZO (a-c), PVDF-HFP (d-f), and PVDF-HFP (w/o LiTFSI) (g-i).

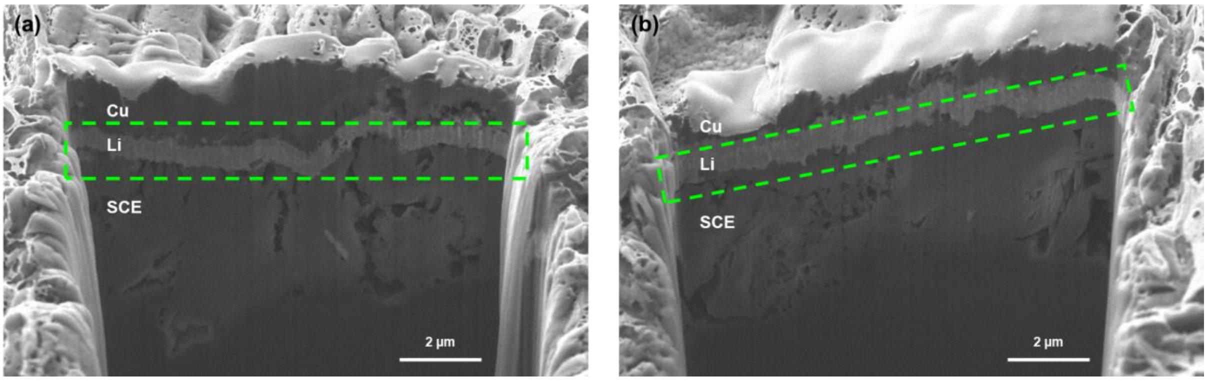

Figure S8. FIB-SEM cross-section images of a) without plasma treatment and b) with plasma treatment.

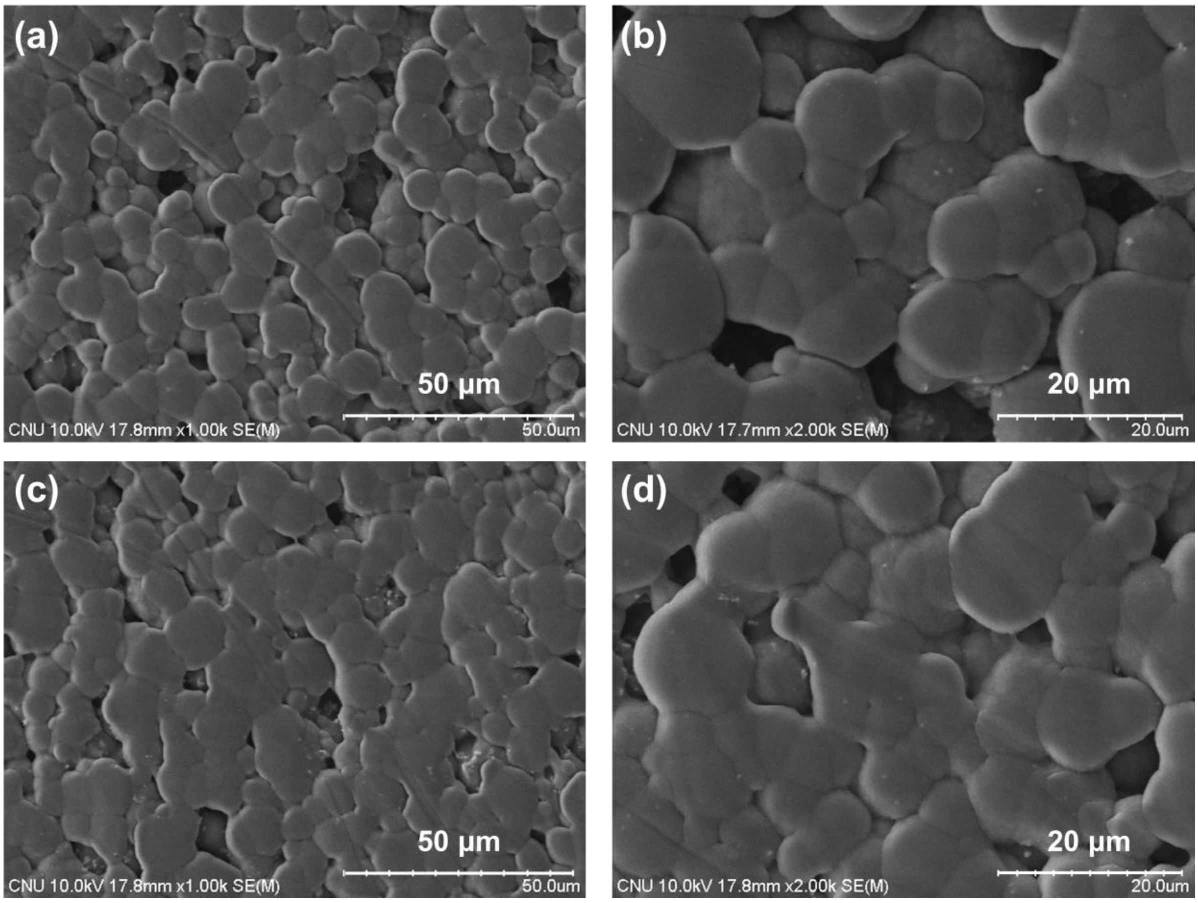

Figure S9. The surface SEM images of a, b) before plasma treatment SCE and c, d) after plasma treatment SCE.

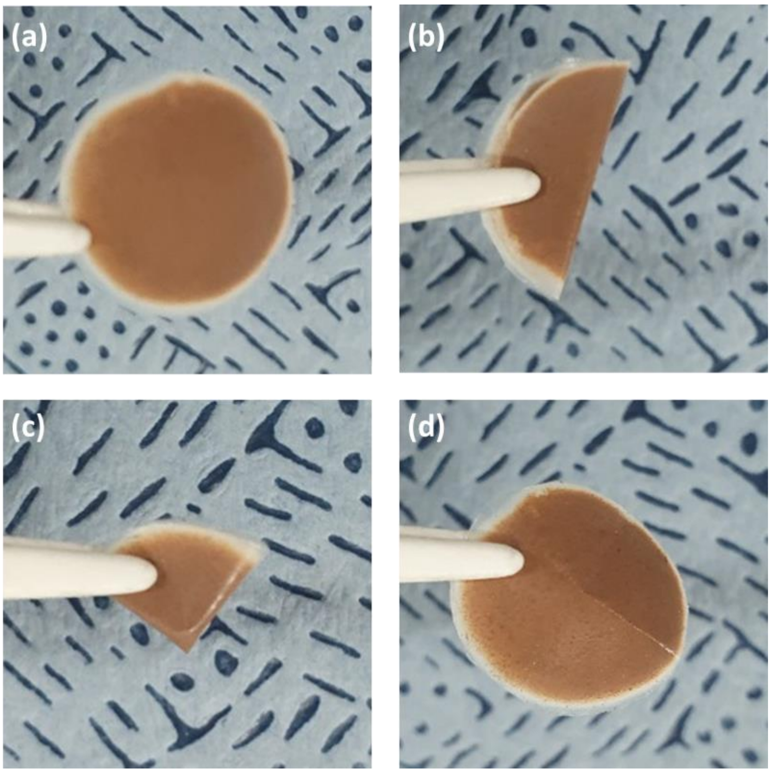

Figure S10. Photograph of flexible and bendable SCE.
